# ^18^F-FDG positron emission tomography and diffusion-weighted magnetic resonance imaging for response evaluation of nanoparticle-mediated photothermal therapy

**DOI:** 10.1038/s41598-020-64617-w

**Published:** 2020-05-05

**Authors:** Marina Simón, Jesper Tranekjær Jørgensen, Kamilla Norregaard, Andreas Kjaer

**Affiliations:** Dept. of Clinical Physiology, Nuclear Medicine & PET and Cluster for Molecular Imaging, Dept. of Biomedical Sciences, Rigshospitalet and University of Copenhagen, Copenhagen, Denmark

**Keywords:** Biophysics, Biotechnology, Cancer, Oncology, Nanoscience and technology, Physics

## Abstract

Nanoparticle-mediated photothermal cancer therapy (PTT) is a treatment which creates localized damage to tumors via nanoparticles that generate heat when irradiated with near infrared light. Substantial work has been dedicated to developing efficient heat-transducing nanoparticles that can be delivered systemically to the tumor. However, less attention has been given to clinically relevant assessment methods of treatment outcome that could be used for personalizing the therapy. Here, we compare ^18^F-FDG positron emission tomography combined with computed tomography (PET/CT) and diffusion-weighted imaging (DWI) for early evaluation and prognosis of PTT in tumor-bearing mice using silica-gold nanoshells (NS). The NS-treated mice experienced inhibited tumor growth and significantly prolonged survival compared to control mice. One day after PTT, PET/CT and DWI scans showed a decrease in tumor ^18^F-FDG uptake of ~90% and an increase of ~50% in apparent diffusion coefficient (ADC) values respectively, compared to baseline. No significant changes were observed for control groups. Additionally, the changes in ^18^F-FDG uptake and ADC values correlated significantly with survival, demonstrating that both methods can be used for early evaluation of PTT although ^18^F-FDG PET/CT showed the strongest prognostic value. Based on these results, both modalities should be considered for therapy monitoring of PTT when clinically translated.

## Introduction

The ability to evaluate if a cancer patient is likely to benefit from a therapy early after initiation is a key element in improving therapy outcome, including survival, as it helps tailoring the appropriate treatment plan for the patient. A commonly used approach for treatment response evaluation is based on morphological changes obtained with anatomical imaging such as computed tomography (CT) or magnetic resonance imaging (MRI). However, a morphological response can be a slow process and can therefore often not be used as an early measure of outcome. As alternatives, non-invasive functional imaging techniques that provide information of the current physiological state of the tumor have been explored, e.g. positron emission tomography (PET). PET has in fact already established itself as an incredibly valuable tool for early diagnosis, staging and follow up of cancer^1–3^. It is a modality where a tracer based on a tumor-relevant biomarker can reveal early treatment-induced changes by comparison of the tracer uptake before and after therapy.

Photothermal cancer therapy (PTT) is a relatively new treatment whose clinical development would benefit from better monitoring and evaluation. In PTT, light-absorbing nanoparticles are used to inflict tumor death through local hyperthermia when irradiated with near infrared (NIR) light^4,5^. Although the therapy has shown to be effective in various preclinical models^6–8^, the outcome is variable as it depends on several biological factors, influencing amongst others the delivery of nanoparticles to the tumor. Therefore, the treatment should be tailored to the individual patient and if the response is absent, the treatment plan should be changed. So far, the outcome of PTT in preclinical studies has mostly been assessed by repeated tumor size measurements with a caliper. However, as previously mentioned, the reduction in tumor volume usually occurs at later stages in clinical settings, and therefore does not really reflect the early treatment response^9^.

Recently we have shown that PET/CT holds prognostic value for early treatment evaluation of PTT in tumor-bearing mice^10–12^.In particular, the PET tracer ^18^F-fluorodeoxyglucose (^18^F-FDG) has been successfully used in cancer imaging since many tumors show an increased rate of glycolysis (Warburg effect) compared to healthy tissue^13^. In our previous studies on PTT, we found that a decrease in ^18^F-FDG uptake in the tumor after treatment could be interpreted as a loss of tumor viability and correlated with improved survival. The use of ^18^F-FDG evaluation can, however, be impaired by tumors that have a low baseline uptake or reside in or in the vicinity of tissues that have a naturally high glucose metabolism. Furthermore, an inflammatory response is also associated with increased ^18^F-FDG uptake that may also hamper tracer specificity^14^.

Diffusion weighted imaging (DWI) is an MR technique that characterizes diffusion of water molecules within tissues^15,16^ and has been used clinically for evaluating diseases such as acute stroke^17^. Recently, it has also been explored for early response monitoring of PTT^18–20^. The motion of water molecules depends on the cell and tissue structure. Accordingly, the high cellularity that characterizes tumor tissue leads to lower water diffusion compared to normal tissue^9^. Furthermore, water diffusion is perturbed in response to therapy-induced changes in the integrity of the tumor tissue. DWI is quantified by the apparent diffusion coefficients (ADC), which are normally found to increase when tissue barriers responsible for restricting water motion are broken down, as is the case in acute necrosis found following PTT.

Hence, the goal of this research was to evaluate and compare the use of ^18^F-FDG PET/CT and DWI for early treatment response monitoring of PTT in tumor-bearing mice.

## Results

### PTT treatment outcome in tumor-bearing mice

In this study, tumor-bearing mice were injected with silica-gold nanoshells (NS), which are nanoparticles already used in clinical trials on PTT^21–23^. Moreover, they have shown to be non-toxic and to have high photothermal efficiency^5,24,25^. CT26 (murine colon carcinoma) was the tumor model of choice due to its extensive use in PTT research^26,27^.

Before treatment, subcutaneous CT26 tumor-bearing mice were divided into three groups based on their tumor volume to match a mean size of ~120 mm^3^ in each group. The three groups represented a NS group (mice receiving NS and NIR laser treatment, n = 7), a saline group (mice receiving saline and laser treatment, n = 7), and a sham group (mice receiving NS but no laser treatment, n = 5). A day after injection of either NS or saline, both the NS and the saline group were irradiated with a laser on the tumor region with an intensity of 2 W/cm^2^ for five minutes (day 0).

**Figure 1:**
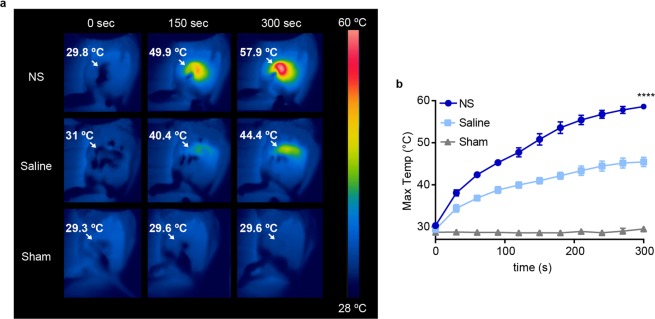
Temperature changes on tumor surface during laser irradiation. (**a**) Representative thermographic images of NS, saline, and sham-treated tumors at three different time points during laser treatment. White arrows point to the tumors and the maximum temperature measured in the frame is given. (**b)** Maximum temperature on the tumor surface during the treatment. Data shown is mean ± SEM. (standard error of the mean). **** denotes *p* < 0.0001 vs. both saline and sham. NS group; n = 7, Saline group; n = 7, sham group; n = 5.

During irradiation, temperatures on the tumor surface were measured using real time thermographic imaging (see representative images in Fig. 1a). The NS group reached a mean maximum temperature of 58.6 ± 0.64 °C on the tumor surface, corresponding to an increase of 28.3 °C (Fig. 1b). In the saline group, the mice also experienced a temperature increase on the tumor surface, but it was only of 16 °C, reaching a mean maximum temperature of 45.4 ± 1.11 °C. As expected, no temperature changes were detected for the sham mice.

**Figure 2:**
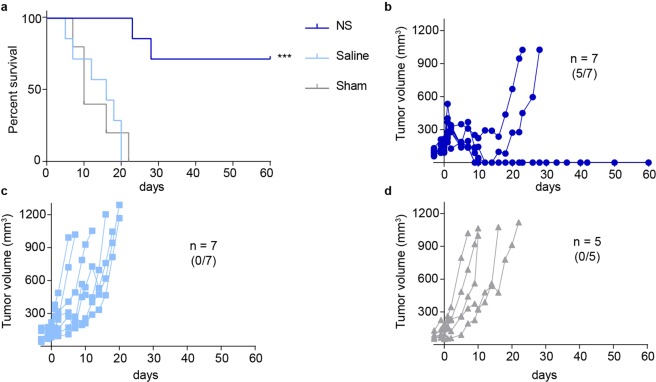
Evaluation of PTT by tumor growth and survival. (**a**) Overall survival for NS (n = 7), saline (n = 7) and sham (n = 5) groups. *** denotes *p* value <0.001 vs. both saline and sham groups. (**b**) Tumor growth for the NS group. 5 out of 7 mice experienced tumor regression up until day 60, when the study was terminated. (**c,d)** Tumor growth curves for saline and sham groups, respectively. No mice experienced tumor regression in these groups.

After treatment, tumor growth was followed using caliper measurements until the tumors either reached a size of 1,000 mm^3^ or until day 60 when the study was terminated. In the NS group, the treatment had a significant impact on survival with five out of seven mice experiencing complete tumor disappearance around one week after treatment and no signs of tumor recurrence 60 days later (undefined median survival). This was in contrast to a median survival of only 10 days for the sham group, and a hazard ratio (HR) of NS vs. sham group of 0.11 (95% CI = 0.01773–0.6611, *p* = 0.0003, Fig. 2a).

As for the saline group, survival was not significantly different from the sham group even though we observed a temperature increase of 16 °C during laser treatment, and tumor growth appeared unaffected. This was likely due to the laser causing unspecific heating on the tumor surface but not an even distribution of the heat throughout the tissue, which in turn did not have an effect on survival. The median survival was 16 days (HR in reference to the sham group = 1.01, 95% CI = 0.3–3.2, *p* = 0.95).

Accordingly, tumor growth was substantially inhibited for the NS group (Fig. 2b) compared to the two control groups (Fig. 2c,d).

### ^18^F-FDG PET/CT for early evaluation of PTT

**Figure 3:**
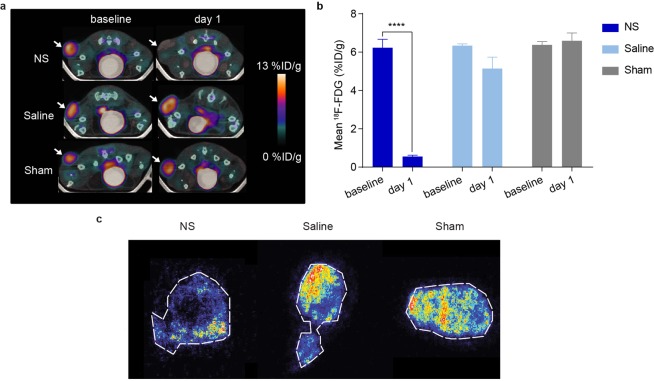
^18^F-FDG PET/CT for early evaluation of PTT. (**a**) Representative ^18^F-FDG PET/CT images of NS (n = 7), saline (n = 7) and sham (n = 5) treated mice before and after PTT. White arrows point to the tumors. (**b**) The mean ^18^F-FDG tumor uptake at baseline and day 1. **** denotes *p* value <0.0001. Data shown is mean ± SEM. (**c)** Autoradiography of NS, saline and sham tumors one day after PTT (n = 2 for each group).

^18^F-FDG PET/CT and DWI were both performed a day before (baseline) and a day after (day 1) PTT in order to be able to compare their value. Mice were PET/CT scanned one hour after intravenous injection of ~10 MBq ^18^F-FDG. From visual inspection of the fused PET/CT images, a clear reduction in ^18^F-FDG uptake was evident in the tumors in the NS group after PTT, whereas the uptake in the tumors in the control groups appeared unchanged (Fig. 3a). The tumor uptake was quantified by whole tumor volume analysis and extracted as mean percent injected dose per gram of tissue (%ID/g). Following treatment, there was a ~90% reduction in mean ^18^F-FDG uptake in the NS group (Fig. 3b). The mean ^18^F-FDG uptake went from 6.2 ± 0.4%ID/g at baseline to 0.55 ± 0.07%ID/g at day 1 (*p* < 0.0001). This great reduction fits well with the significantly prolonged survival in the NS group.

For the mice in the saline and sham groups, there was no significant decrease in the mean tumor uptake of ^18^F-FDG (from 6.3 ± 0.09%ID/g at baseline to 5.1 ± 0.6%ID/g at day 1; *p* = 0.0691 in the saline group, and from 6.4 ± 0.17%ID/g to 6.6 ± 0.41%ID/g; *p* = 0.9757 in the sham group).

On day 1 after PTT, autoradiography was also performed to examine tracer distribution in the tumor one hour after ^18^F-FDG injection on tissue sections from all groups. This confirmed that NS-treated tumors had reduced uptake of ^18^F-FDG compared to the saline and sham groups (Fig. 3c).

### Diffusion-weighted imaging for early evaluation of PTT

**Figure 4:**
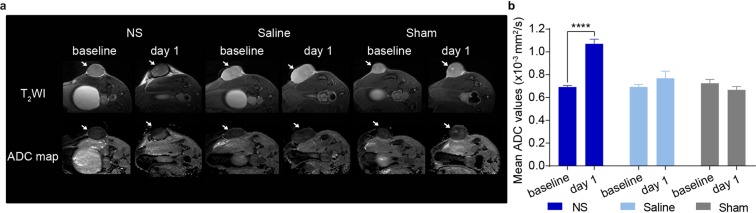
DWI for early evaluation of PTT. (**a**) Representative T_2_WI and ADC maps of NS (n = 7), saline (n = 7) and sham (n = 5) treated animals at baseline and after treatment (day 1). White arrows point to the tumors. (**b**) The mean ADC values at baseline and day 1. **** denotes *p* value <0.0001. Data shown is mean ± SEM.

Right after the ^18^F-FDG PET/CT scan, we performed MRI. On the MR images we observed signal changes after PTT on theT_2_-weighted and DW images from the tumors in the NS group (Fig. 4a). We also observed the presence of edema around the tumor area, caused by PTT. In comparison, there was no clear change after treatment in the tumors from the saline and sham groups. Figure 4b shows the ADC values obtained from the DWI. Before PTT, the mean ADC was 0.69 ± 0.01 × 10^−3^ mm^2^/s in the NS group whereas it increased to 1.07 ± 0.04 × 10^−3^ mm^2^/s one day after therapy (*p* < 0.0001). This change corresponds to a significant increase of around 50%.

In contrast, this increment was not observed in the mean ADC values found for the saline group (0.69 ± 0.02 ×10^−3^ mm^2^/s at baseline to 0.77 ± 0.06 × 10^−3^ mm^2^/s at day 1, *p* = 0.2309), or the sham group (0.72 ± 0.03 ×10^−3^ mm^2^/s at baseline and 0.67 ± 0.03 × 10^−3^ mm^2^/s at day 1, *p* = 0.5737, Fig. 4b).

### Immunohistochemical detection of PTT-induced tumor damage

**Figure 5:**
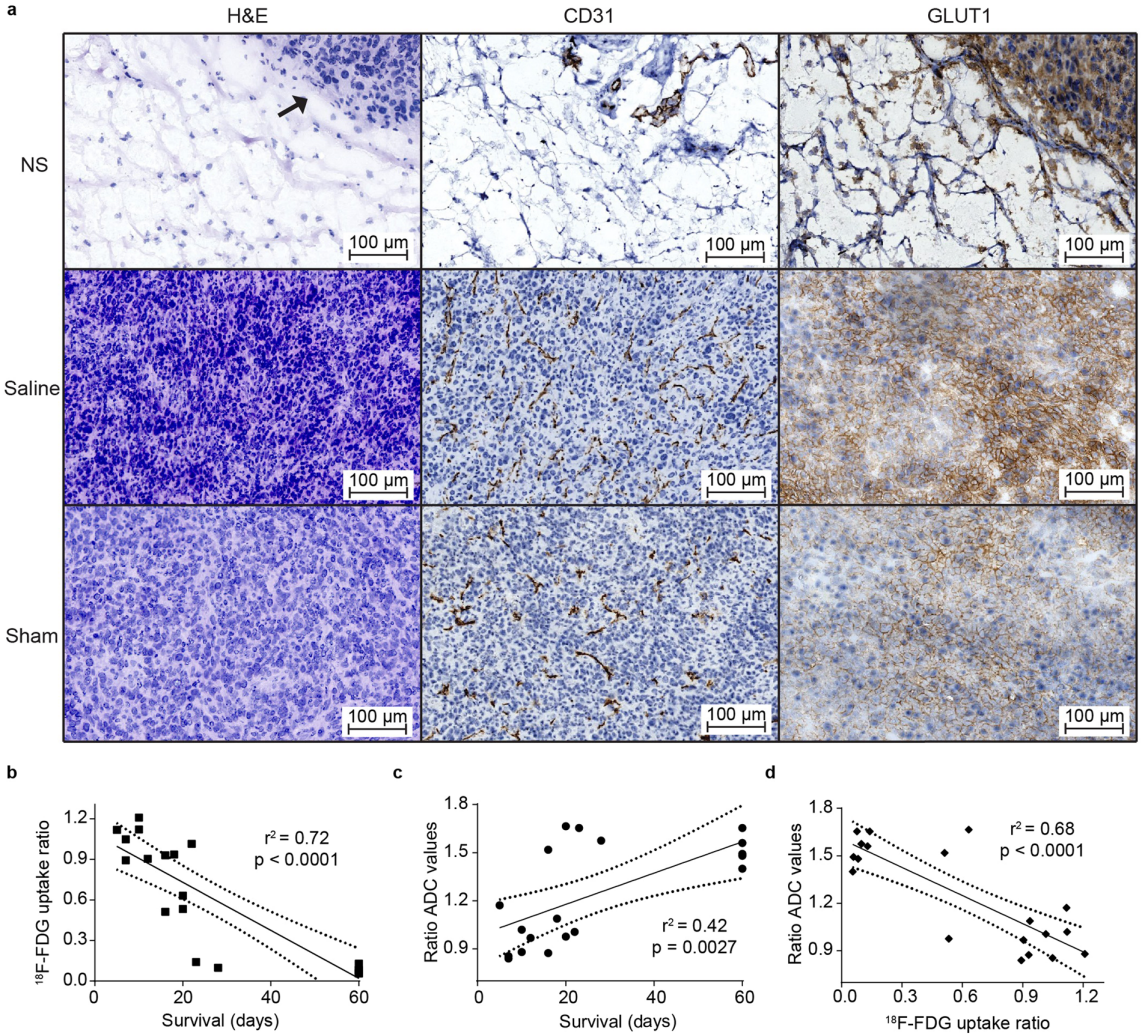
Correlation between treatment-induced changes in the^18^F-FDG uptake and ADC values and survival length. (**a**) Representative images of the H&E (left), CD31 (middle), and GLUT1 (right) staining of tumors from the NS, saline and sham groups one day after PTT (n = 2 for each group). In the H&E staining, the arrow on the NS tumor points to the border between healthy and necrotic tumor tissue. The brown color indicates CD31 and GLUT1 positive cells. (**b)** Correlation between the ratio of ^18^F-FDG uptake and days of survival. (**c)** Correlation between the ratio of ADC values and days of survival. (**d)** Correlation between the ratio of ADC values and ^18^F-FDG uptake. (**b–d)** n = 19.

To verify that changes in ^18^F-FDG tumor uptake and ADC values after PTT were caused by treatment-induced tumor damage, we performed histological analysis on tumor tissue sections from all groups resected one day after treatment. Hematoxylin and eosin (H&E) and CD31 staining are markers for cell viability and tumor angiogenesis, respectively. As expected, large regions of the tumor in the NS group displayed severe cell death and highly irregular tissue structure visualized by H&E staining (Fig. 5a). This was not observed in any of the two control groups. CD31 staining showed that the vasculature in NS tumors was completely damaged and positive staining was only found in small regions. Saline and sham-treated tumors presented intact vasculature throughout the whole tumor tissue. Furthermore, immunohistochemical staining towards Glucose Transporter 1 (GLUT1) was also conducted as it can be used as a biomarker for ^18^F-FDG uptake. High levels of GLUT1 expression were observed in saline and sham treated tumors throughout the entire tumor mass. In contrast, NS treated tumors exhibited clearly reduced levels of GLUT1 and staining could only be found in small regions where viable tissue remained. Hence, the expression of GLUT1 observed with immunohistochemical staining was consistent with the ^18^F-FDG uptake observed in the PET scans.

### Correlation between molecular imaging and survival

To evaluate the ability of the two techniques to predict treatment outcome, the change in signal after treatment compared to baseline (i.e., ratio of day 1/baseline) was plotted against days of survival. For the ^18^F-FDG uptake ratio, we detected a strong, negative correlation with survival (r^2^ of 0.72 and *p* < 0.0001; Fig. 5b). In contrast, the ratio of ADC values only showed a moderate positive correlation with survival (r^2^ of 0.42 and *p* = 0.0027; Fig. 5c). Hence, for PTT evaluation we found that ^18^F-FDG PET/CT had a stronger predictive value than DWI. However, there was still a strong correlation (r^2^ of 0.68 and *p* < 0.0001) between the change in ^18^F-FDG uptake and the change in ADC values obtained after treatment (Fig. 5d), indicating that both modalities are not independent and both can be used to obtain information of the treatment outcome.

## Discussion

In preclinical studies in tumor-bearing mice, PTT has proven to be able to eradicate tumor tissue while minimizing damage to healthy surrounding tissue. Nanoparticles such as NS can efficiently accumulate in the tumor and transform light into heat, leading to localized and irreversible damage. However, many factors including laser dose and tumor characteristics such as size and vasculature, influence the response and therefore there is often a variation in treatment outcome between individuals^28,29^. Hence, from a translational point of view, optimization and personalization of the therapy is needed. For this, imaging techniques that are able to predict long-term treatment response early after therapy are of great interest.

Here, we used PET/CT imaging and DWI for early evaluation of PTT by analysis of the changes in tumor ^18^F-FDG uptake and ADC values, respectively. Post-treatment scans were performed one day after PTT since our previous study showed that significant changes in ^18^F-FDG uptake could be detected^10^ and Zhang *et al*.^18^ reported a peak in ADC values at this time point. The tumors that received NS and laser irradiation responded very well and five out seven mice were tumor free 60 days after treatment, when the study was terminated. Both ^18^F-FDG PET/CT and DWI showed significant changes in the tumor after therapy as a result of loss of cell viability and the disruption of cellular barriers, confirmed by H&E, CD31, and GLUT1 staining. On the day after therapy, ^18^F-FDG uptake was reduced to less than 10% of its baseline value whereas the ADC values increased by ~50%. No significant changes were found in ^18^F-FDG uptake, ADC values or histological analysis of tissue sections in either of the control groups.

In addition, we found that the change in ^18^F-FDG uptake correlated strongly with survival whereas the change in ADC value only correlated moderately with survival, indicating that ^18^F-FDG PET/CT is likely to perform better than DWI for early treatment evaluation of PTT. However following therapy, the change in ^18^F-FDG uptake correlated well with the inverse change in ADC values (r^2^ of 0.68 and *p* < 0.0001) meaning that tumors with high decrease in ^18^F-FDG uptake also had a high increase in ADC values. Accordingly, these results imply that both techniques are predictive of long-term treatment outcome and that DWI may be a good alternative in cases where ^18^F-FDG PET/CT is unavailable or cannot be used.

Limitations to the use of ^18^F-FDG are, among others, that it does not only accumulate in cancer tissue. High levels of the tracer can be seen in heart, kidneys, brain and bladder^30^. Furthermore, not all tumors show increased glucose uptake, and non-cancerous inflammation can also cause a higher ^18^F-FDG uptake and lead to false positive results^14^. Limitations to DWI are the poor spatial resolution and the fact that it is highly sensitive to motion artifacts, e.g., tumors close to the respiratory system or heart, which can hinder treatment assessment. Also inflammation can lead to decreased diffusion and wrong interpretation of the results^31,32^.Moreover, there is a lack of standardized procedures for image analysis. The timing of post-treatment scans should also be considered as studies have shown that the ADC values vary quite dramatically within the first days after PTT^18,19^. DWI has the advantage over ^18^F-FDG PET/CT that it does not involve radiation exposure or require contrast agents that are injected systemically, and the examination time is faster.

Finally, the two modalities could also be considered as complementary to each other since the information they provide is representing different biological functions.

To conclude, in this study of NS-mediated photothermal therapy in mice bearing subcutaneous tumors we demonstrated that both ^18^F-FDG PET/CT and DWI could be used as early measures of treatment effect. Furthermore, the results suggest that changes in ^18^F-FDG uptake and ADC values are useful prognostic tools that can be utilized to guide PTT, although ^18^F-FDG PET/CT holds stronger promise than DWI and therefore should be chosen if available.

## Materials and methods

### Nanoparticles

800 nm Resonant BioPure Gold NS were purchased from NanoComposix, USA. The total diameter of the particles was 157 ± 9 nm, measured with Transmission Electron Microscopy (TEM) by the supplier, and the diameter of the silica core was 119 nm ± 5 nm. The NS were functionalized with 5 kDa poly(ethylene glycol) and the zeta potential was reported as −42 mV.

### Animal model

The animal experiments were approved by the Danish Animal Welfare Council, Ministry of Justice. All methods and experiments were performed in accordance with the relevant guidelines and regulations. The animals were 5-week-old BALB/cJRj female mice from Janvier Labs (Le Genest Saint Isle, France). They were allowed to acclimatize for a week after arrival before starting the experiments. For tumor inoculation, CT26 cells (ATCC) were first cultured in RPMI 1640 + *GlutaMAX* medium with 10% fetal bovine serum and 1% penicillin-streptomycin (Thermo Fisher Scientific) at 37 °C and in 5% CO_2_. When the cells reached around 70% confluence, 3 × 10^5^ cells (in 100 µl of PBS) were injected into the left flank of the mice. The mice were kept under anesthesia throughout the inoculation procedure by breathing 3–5% sevoflurane (Abbott Scandinavia AB, Sweden) mixed with 35% O_2_ in N_2_. Tumor size was measured every two or three days with the use of a caliper and the tumor volume calculated as: volume = ½ (length × width^2^). A tumor volume of 1,000 mm^3^ was used as the humane endpoint and animals that experienced complete tumor regression were euthanized on day 60, when the study was terminated.

### Photothermal therapy

Mice were injected with 190 μl of either NS (5 × 10^10^ NS/ml) or saline through the tail vein at the day of the baseline scan. Here, mean tumor size was 127.9 mm^3^ in the NS group (n = 7), 119.6 mm^3^ in the saline group (n = 7), and 122.7 mm^3^ in the sham group (n = 5). The following day, the animals were anesthetized by breathing sevoflurane and placed on a heating pad placed below an 807 nm diode laser beam (beam diameter of ~1 cm). The laser intensity used was 2 W/cm^2^. During the 5-minute laser irradiation, the maximum temperature on the tumor surface was recorded using real-time thermographic imaging (FLIR T-440 camera), and pictures were taken every 30 seconds. Right before the treatment, glycerol was swabbed onto the tumors to facilitate light penetration and reduce scattering through the skin^12,33–35^. Sham mice did not receive laser irradiation but were placed under the laser for five minutes and tumors were swabbed with glycerol to mimic the conditions. Temgesic (0.3 mg/ml) was used for pain relief and injected subcutaneously before the treatment and every six to eight hours until deemed necessary to avoid unnecessary distress. FLIR images were analyzed within the FLIR Tools software.

### PET/CT

^18^F-FDG was produced at the Department of Clinical Physiology, Nuclear Medicine and PET, Rigshospitalet, Centre of Diagnostic Investigations, Copenhagen, Denmark. Approximately 10 MBq of ^18^F-FDG were injected into the tail vein one hour before the PET scan. ^18^F-FDG PET scans were performed on a MicroPET Focus 120 scanner (*Concorde* Microsystems Inc., Knoxville, TN, USA) and CT scans were performed with a nanoScan SPECT/CT scanner (Mediso Medical Imaging Systems, Budapest, Hungary). Mice were kept anesthetized with sevoflurane during all scan procedures and their temperature kept stable using a heating pad. The parameters used for the CT scan were, as described previously^12^, 720 projections, 300 ms of exposure time and 35 kVp of x-ray energy. The parameters for the PET scan were 300 s of acquisition time, energy window of 350–650 keV and a timing window of 6 ns. PET data was processed into sinograms and reconstructed using the maximum a posteriori algorithm. PET and CT images were manually fused in the Inveon software (Siemens Medical Solutions) and the ^18^F-FDG uptake in the tumors measured by manually drawing a region of interest (ROI) on the whole tumor.

### MRI

Following PET/CT, the mice were transferred to a Bruker PharmaScan 7 T Small Animal MRI Scanner. Animals were kept anesthetized as described before and placed on the side, so a surface coil could be positioned on top the tumor. For T_2_WI a TurboRare sequence was used with the parameters TR = 2500 ms, TE = 33 ms, field of view = 30 × 30 mm, matrix = 256 ×256, slice thickness = 1 mm, 13 contiguous slices, averages = 2. For DWI: TR = 2000 ms, TE = 27 ms, field of view = 30 × 30 mm, matrix = 128 × 128, slice thickness = 1 mm, 13 contiguous slices, averages = 6, b values = 0, 1000 s/mm^2^.

ROIs were drawn on the tumor using the Free DICOM Medical Image Viewer Horos. ADC values were obtained from each ROI. ADC maps were obtained for all groups. The threshold was set at 4% (of maximum in b = 0 image).

### Autoradiography and histological analysis

A day after PTT, animals from the NS, saline and sham groups (n = 2 in each group) were injected with ^18^F-FDG and anesthetized for 1 hour. After that period, animals were euthanized and tumors resected. Tumors were snap-frozen by submerging them into isopentane and thereafter embedded in tissue-tek. Subsequently, they were cut into 8 µm thick slices using a Leica CM1850 UV cryostat (Leica Biosystems, Germany) that were exposed for 20 minutes on phosphorous films and imaged on Amersham Typhoon Biomolecular Imager (GE Healthcare). Autoradiography images were analyzed within the ImageQuant TL v8.1 Image Analysis Software. Tissue slides were left to dry overnight and were transferred to −80 °C the day after for storage until staining.

Slides were brought to room temperature, fixated in 4 °C acetone, and submerged in HistoClear solution to ensure complete removal of tissue-tek. For H&E, they were stained with hematoxylin for 5 minutes, rinsed and stained with eosin for 3 minutes.

To perform the immunostaining, heat-induced epitope retrieval was performed by covering the tissue slides with 250 mL citrate buffer (pH = 6) for GLUT1 and Tris Buffer (pH = 9) for CD31, while steaming at 100 °C for 15 minutes in a microwave. The slides were then left to equilibrate to room temperature and rinsed in running water. Following this, the slides were submerged in phosphate buffered saline with KCl (K-PBS) + tween 20 (137 mM NaCl, 2.7 mM KCl, 0.1% Tween20) for five minutes and afterwards transferred to K-PBS. For the actual staining procedure, the slides were placed horizontally in a humidified chamber. The slides were first blocked with Peroxidase-Blocking Solution (Dako; S2023) for 10 minutes, and then with bovine serum albumin, BSA, (2% BSA in K-PBS, Sigma Aldrich; A79096) for 20 minutes. Primary antibodies were diluted in BSA and incubated for 1 hour at room temperature. The antibodies used were Anti-GLUT1 (1:1500, Abcam; ab652) and Anti-CD31 (1:250, Abcam; ab28364). Following, the slides were incubated with HRP (horseradish peroxidase)-labeled polymer conjugated to secondary antibody (EnVision + System-HRP Labeled Polymer) anti-rabbit for 40 minutes at room temperature. This was followed by incubation with DAB (Liquid DAB + Substrate System TM, Dako; K3468) for five minutes. The sections were subsequently counterstained with Mayers acidic hematoxylin (Region Hovedstadens Apotek, 854183) for one minute. The slides were mounted using an automated glass coverslipper (Dako). Sections were scanned on Zeiss Axio Scan.Z1.

### Data analysis and statistics

Survival curves were created using the Kaplan-Meier method and compared through the log-rank test. Hazard ratios (HR) were calculated using the log-rank test. The mean ^18^F-FDG uptake and mean ADC values were compared using two-way ANOVA with Sidak’s multiple comparisons test. The maximum temperatures were compared using one-way ANOVA with Tukey’s multiple comparisons test. The correlation graphs were calculated using linear regression and show the 95% confidence bands of the best-fit line. The data was plotted in Prism7 and shown as mean ± SEM.
